# Release of STK24/25 suppression on MEKK3 signaling in endothelial cells confers cerebral cavernous malformation

**DOI:** 10.1172/jci.insight.160372

**Published:** 2023-03-08

**Authors:** Xi Yang, Shi-Ting Wu, Rui Gao, Rui Wang, Yixuan Wang, Zhenkun Dong, Lu Wang, Chunxiao Qi, Xiaohong Wang, M. Lienhard Schmitz, Renjing Liu, Zhiming Han, Lu Wang, Xiangjian Zheng

**Affiliations:** 1 Department of Pharmacology and Tianjin Key Laboratory of Inflammation Biology, School of Basic Medical Sciences, and Center for Cardiovascular Diseases, Tianjin Medical University, China.; 2 State Key Laboratory of Experimental Hematology, Institute of Hematology and Blood Diseases Hospital, Chinese Academy of Medical Sciences and Peking Union Medical College, Tianjin, China.; 3 Institute of Biochemistry, Justus Liebig University, Member of the German Center for Lung Research, Giessen, Germany.; 4 Vascular Epigenetics Laboratory, Victor Chang Cardiac Research Institute, and St. Vincent’s Clinical School, University of New South Wales, Sydney, Australia.; 5 State Key Laboratory of Stem Cell and Reproductive Biology, Institute of Zoology, Chinese Academy of Sciences, and; 6 Beijing Institute for Stem Cell and Regenerative Medicine, Beijing, China.

**Keywords:** Angiogenesis, Vascular Biology, Endothelial cells, Genetic diseases, Stroke

## Abstract

Loss-of-function mutations in cerebral cavernous malformation (CCM) genes and gain-of-function mutation in the *MAP3K3* gene encoding MEKK3 cause CCM. Deficiency of CCM proteins leads to the activation of MEKK3-KLF2/4 signaling, but it is not clear how this occurs. Here, we demonstrate that deletion of the CCM3 interacting kinases STK24/25 in endothelial cells causes defects in vascular patterning during development as well as CCM lesion formation during postnatal life. While permanent deletion of STK24/25 in endothelial cells caused developmental defects of the vascular system, inducible postnatal deletion of STK24/25 impaired angiogenesis in the retina and brain. More importantly, deletion of STK24/25 in neonatal mice led to the development of severe CCM lesions. At the molecular level, a hybrid protein consisting of the STK kinase domain and the MEKK3 interacting domain of CCM2 rescued the vascular phenotype caused by the loss of *ccm* gene function in zebrafish. Our study suggests that CCM2/3 proteins act as adapters to allow recruitment of STK24/25 to limit the constitutive MEKK3 activity, thus contributing to vessel stability. Loss of STK24/25 causes MEKK3 activation, leading to CCM lesion formation.

## Graphical abstract

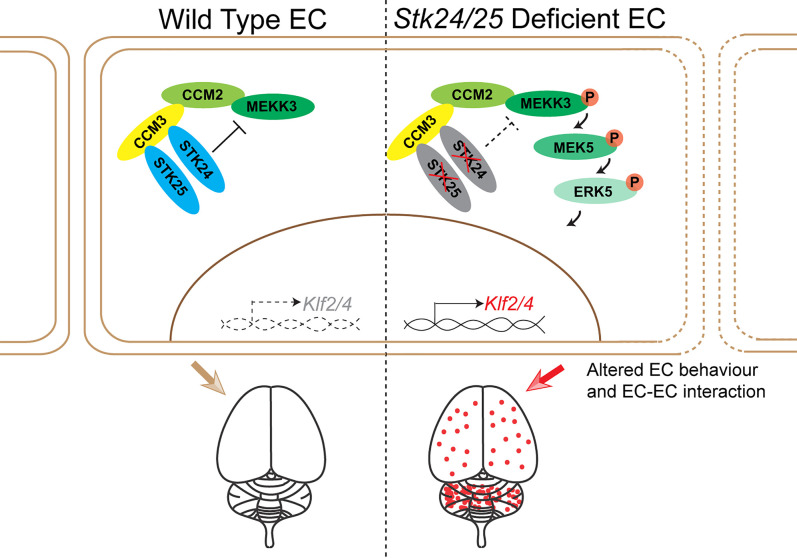

## Introduction

Mutations in the cerebral cavernous malformation (CCM) genes, *KRIT1*, *CCM2*, and *PDCD10*, cause CCM disease. The CCM genes encode KRIT1 (also referred to as CCM1), CCM2, and PDCD10 (also referred to as CCM3) proteins that act as adapter proteins and can form a single signaling complex (1, 2). Biochemical studies revealed that CCM1 interacts with CCM2 and that CCM2 interacts with CCM3 (3–6). The interaction with CCM1 induces a conformation change in CCM2 and enhances its interaction affinity with CCM3 (4). Mutations that disrupt CCM complex formation causes human diseases (7, 8). The CCM complex also interacts with other proteins; CCM2 interacts with MEKK3 and Rac, whereas CCM3 can complex with the kinases STK24/25 and MST4, which belong to the germinal-center kinase III (GCKIII) subfamily of kinases (4, 5, 9). These kinases appear to initiate 2 downstream signaling pathways, namely the MEKK3-KLF2/4 and STK24/5-Ezrin/Radixin/Moesin (STK24/5-ERM) signaling pathways (4, 10, 11). Loss of CCM genes leads to elevated MEKK3-KLF2/4-ADAMTS4/5 signaling (10, 11). A causative role of this elevated signaling pathway leading to the development of cardiovascular defects and CCM lesions was shown by the beneficial effects of pharmacological MEKK3 inhibition or genetic reduction of MEKK3, KLF, or ADAMTS signaling (10–13). In support of this, the recent identification of a gain-of-function mutation in *MAP3K3* in patients with CCM emphasized a causative role of this kinase in CCM pathogenesis (14). In addition, experiments in zebrafish and in cultured HUVECs, suggest that the interaction between STK and CCM3 employs a ERM-RHO signaling pathway to regulate cardiovascular development (4).

In this study, we generated mice with floxed alleles of *Stk24* and *Stk25* to delete both genes in endothelial cells. Defective STK24/25 expression in endothelial cells caused defects in vascular development and CCM lesion formation, akin to the phenotype observed with *Ccm* gene deletions. Biochemical experiments suggest that STK24/25 prevents CCM pathogenesis through restriction of constitutive MEKK3 activity.

## Results

### Deletion of STK24/25 in endothelial cells restricts lumen formation of BAA and the DA.

**Figure 1:**
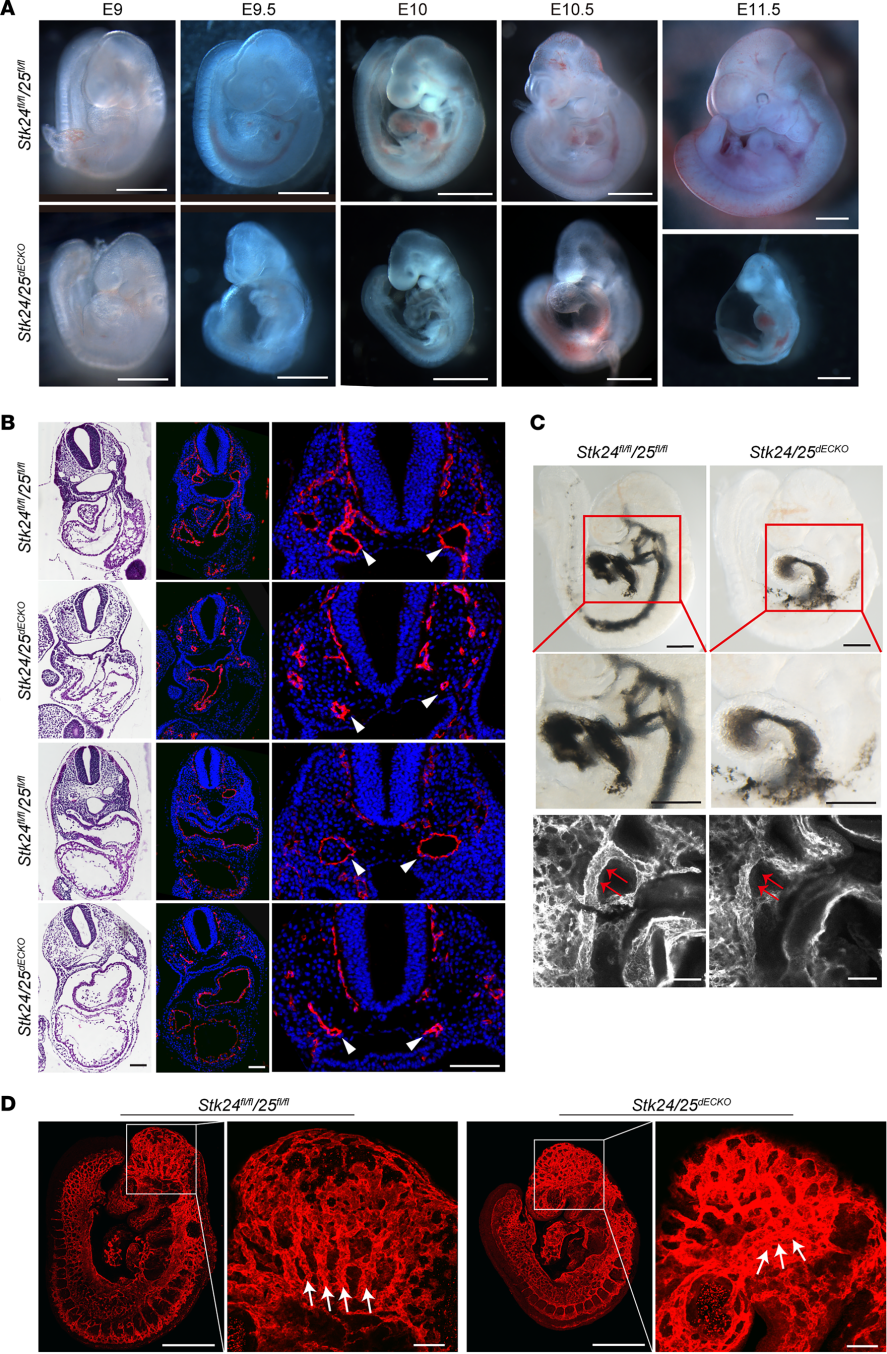
Deletion of *Stk24* and *Stk25* in endothelium results in vascular defects during embryonic development. (**A**) Stereomicroscopic images of developmental time course of littermate *Stk24^fl/fl^;Stk25^fl/fl^* and *Stk24/25^dECKO^* mice. Scale bars: 1 mm. (**B**) H&E staining and Pecam immunostaining of transverse sections of E10 *Stk24^fl/fl^;Stk25^fl/fl^* (*n* = 3) and *Stk24/25^dECKO^* (*n* = 4) embryos reveal the presence of normally lumenized dorsal aortas (DA) in the *Stk24^fl/fl^;Stk25^fl/fl^* embryos but not in *Stk24/25^dECKO^* embryos. White arrowheads indicate dorsal aortas. Scale bars: 100 μm. (**C**) Images of E9.5 embryo hearts of *Stk24^fl/fl^;Stk25^fl/fl^* (*n* = 9) and *Stk24/25^dECKO^* (*n* = 6) embryos with injection of Indian ink. Upper and middle panels show embryo overview and magnification of the boxed regions, showing injected ink flows primarily through the second and third BAA to fill the DA in the *Stk24^fl/fl^;Stk25^fl/fl^* embryos. In contrast, ink injected into the heart of *Stk24/25^dECKO^* embryos failed to opacify the DA. Accumulation of ink was observed in the heart due to the narrow BAA. Scale bars: 500 μm. Lower panel shows whole-mount immunostaining for the endothelial cell marker, endoglin, showing narrowed BAA and adjacent DA (red arrows) in *Stk24/25^dECKO^* embryos. Scale bars: 100μm. (**D**) Whole-mount immunostaining with endoglin showing the impaired vascular patterning (indicated by the white arrows) in the brain of E9.5 *Stk24/25^dECKO^* (*n* = 6) embryos in comparison with that of *Stk24^fl/fl^;Stk25^fl/fl^* (*n* = 3) littermate control embryos. Scale bars: 500 μm in overview panels and 100 μm in magnified panels. All the images presented are representatives of 3 or more independent experiments.

Mass spectrometry and biochemical studies have identified the GCKIII subfamily kinases STK24, STK25, and MST4 as binding partners of CCM3 (4, 5, 15). To determine the role of endothelial STK24 and STK25 in vascular development, we crossed the *Stk24^fl/fl^* and *Stk25^fl/fl^* mice with the *Tie2-Cre* mice to delete the *Stk24* and/or *Stk25* genes in the endothelial lineage. Both the *Tie2-Cre;Stk24^fl/fl^* and *Tie2-Cre;Stk25^fl/fl^* mice were born at expected numbers and without an overt phenotype. Further genetic analyses showed that the simultaneous deletion of *Stk24* and *Stk25* in endothelial cells (the *Tie2-Cre;Stk24^fl/fl^;Stk25^fl/fl^* mice, hereafter referred to as the *Stk24/25^dECKO^*) led to embryonic lethality. Timed mating revealed that the *Stk24/25^dECKO^* embryos died before E11, while *Tie2-Cre;Stk24^fl/fl^;Stk25^fl/+^* and *Tie2-Cre;Stk24^fl/+^;Stk25^fl/fl^* littermates were unaffected and appear grossly normal at E11.5 or E12 (Figure 1A, Supplemental Tables 1 and 2, and Supplemental Figure 1; supplemental material available online with this article; https://doi.org/10.1172/jci.insight.160372DS1). Histologic analysis revealed that *Stk24/25^dECKO^* failed to form patent branchial arch arteries (BAA) and the dorsal aorta (DA) (Figure 1B and Supplemental Figure 2A). This restricted formation of BAA and DA prevent the formation of a functional circulation system. Injection of Indian ink and subsequent analysis of its distribution showed that the injected ink was confined to the heart in the *Stk24/25^dECKO^* embryos at E10 (Figure 1C and Supplemental Figure 2B). Whole-mount staining of endoglin confirmed the restricted DA (Figure 1C) and also showed the mispatterning of brain vasculature (Figure 1D and Supplemental Figure 3). The developmental patterning of BAA, DA, and brain vasculature in mice with deletion of only *Stk24* (*Tie2-Cre;Stk24^fl/fl^;Stk25^fl/+^*) were normal (Supplemental Figures 1–3). These data indicate that double deletion of *Stk24* and *Stk25* is required to induce a vascular phenotype similar to that of *Ccm* gene deficiency (16, 17). These data suggests STK24 and STK25 complement each other and play a critical role in the CCM pathway to regulate the lumenization of the BAA and the DA as well as the patterning of the brain vasculature.

### Induced postnatal deletion of Stk24/25 impairs angiogenesis and confers CCM lesion formation.

**Figure 2:**
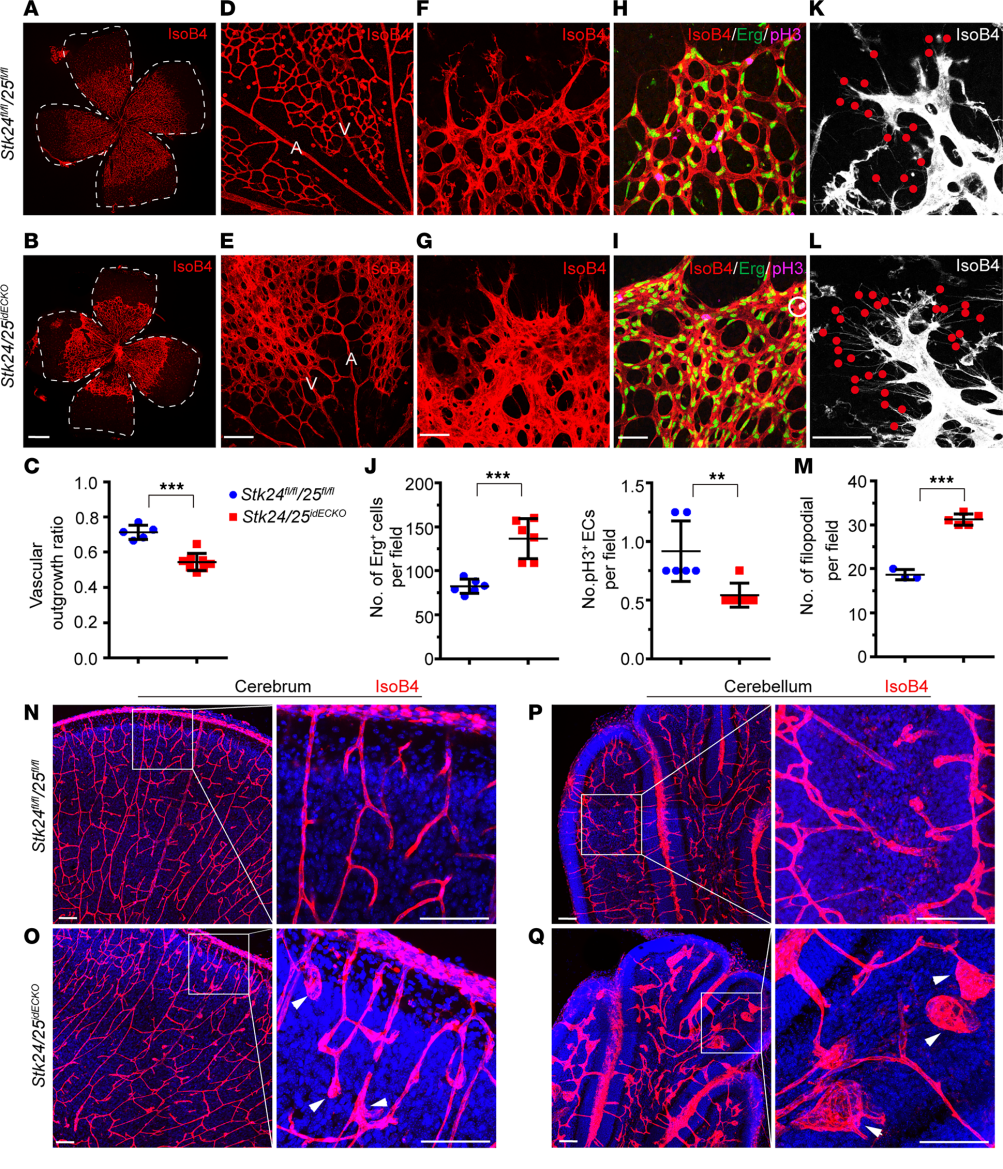
Deletion of *Stk24* and *Stk25* in endothelium of newborn pups disrupt retina vascular extension and filopodia formation. (**A** and **B)** Whole-mount staining of retinal vasculature with IsoB4 in *Stk24^fl/fl^;Stk25^fl/fl^* (*n* = 5) and *Stk24/25^idECKO^* (*n* = 8) mice at P6. The white line highlights the total retinal area. Scale bars: 500 μm. (**C**) Quantitative analysis shows reduced retinal vascular outgrowth in *Stk24/25^idECKO^* mice compared with that of *Stk24^fl/fl^;Stk25^fl/fl^* mice. Each data point represents 1 mouse. (**D**–**G**) IsoB4 whole-mount stainings of P6 retinas show vascular remodeling close to arteries and veins (**D** and **E**) and peripheral vessel plexus (**F** and **G**) in *Stk24^fl/fl^;Stk25^fl/fl^* mice (*n* = 4) and *Stk24/25^idECKO^* mice (*n* = 4). A indicates arteries; V denotes veins. Scale bars: 100 μm. (**H** and **I**) Confocal images of IsoB4, pH3, and Erg costaining of P6 retina in *Stk24^fl/fl^;Stk25^fl/fl^* mice (*n* = 6) and *Stk24/25^idECKO^* mice (*n* = 6). The white circle indicates the pH3^+^ ECs. Scale bars: 100 μm. (**J**) Quantitative analysis shows increased Erg^+^ cells but decreased pH3^+^ ECs in *Stk24/25^idECKO^* mice compared with *Stk24^fl/fl^;Stk25^fl/fl^* mice. Each data point represents 1 mouse. (**K** and **L**) Confocal images of P6 retina lobe stained with IsoB4 in *Stk24/25^idECKO^* mice (*n* = 5) compared with *Stk24^fl/fl^;Stk25^fl/fl^* mice (*n* = 3). The red dots denote filopodia in the vascular front. Scale bars: 100 μm. (**M**) Quantitative analysis showing increased filopodia numbers in *Stk24/25^idECKO^* mice compared with *Stk24^fl/fl^;Stk25^fl/fl^* mice. (**N**–**Q**) Confocal images and magnification of IsoB4 staining in the cerebral cortical vasculature (**N** and **O**) and cerebellum (**P** and **Q**) of P8 mouse pups (*n* = 4 for both *Stk24/25^idECKO^* and *Stk24^fl/fl^;Stk25^fl/fl^*). The white arrows indicate the malformation vessel. Scale bars: 100 μm. All the images presented are representatives of 3 or more independent experiments. The quantitative data are presented as mean ± SD, and significance was determined using unpaired *t* test. ***P < 0.001, **P < 0.01.

Due to the lethality of the *Stk24/25^dECKO^* embryos during early embryonic development, we generated a genetic system that allow for the inducible ablation of these genes. To achieve this, the *Stk24^fl/fl^;Stk25^fl/fl^* mice were crossed with *Cdh5-CreERT2* mice to generate the *Cdh5-CreERT2;Stk24^fl/fl^;Stk25^fl/fl^* mice (denoted as *Stk24/25^idECKO^*). Induction of *Stk24/Stk25* deletion at P2 through intragastric injection of 4-hydroxytamoxifen (4-HT) led to decreased retinal vessel outgrowth (Figure 2, A–C) as well as defective remodeling of the retinal venous branches and peripheral vessel remodeling (Figure 2, D–G). The number of endothelial cells in the malformed areas in the *Stk24/25^idECKO^* mice were increased compared with littermate controls, but the number of proliferatively active cells (as determined by phosphorylated histone 3 [pH3] staining) were not increased (Figure 2, H–J). Tip cells of the retina vasculature of *Stk24/25^idECKO^* mice showed increased filopodia protrusions (Figure 2, K–M, and Supplemental Figure 4). Imaging of the brain vessels in vibratome sections revealed vasculature mispatterning and the presence of small cavernous vessels in the cerebrum and the cerebellum of the *Stk24/25^idECKO^* mice (Figure 2, N–Q, and Supplemental Figures 5 and 6).

**Figure 3:**
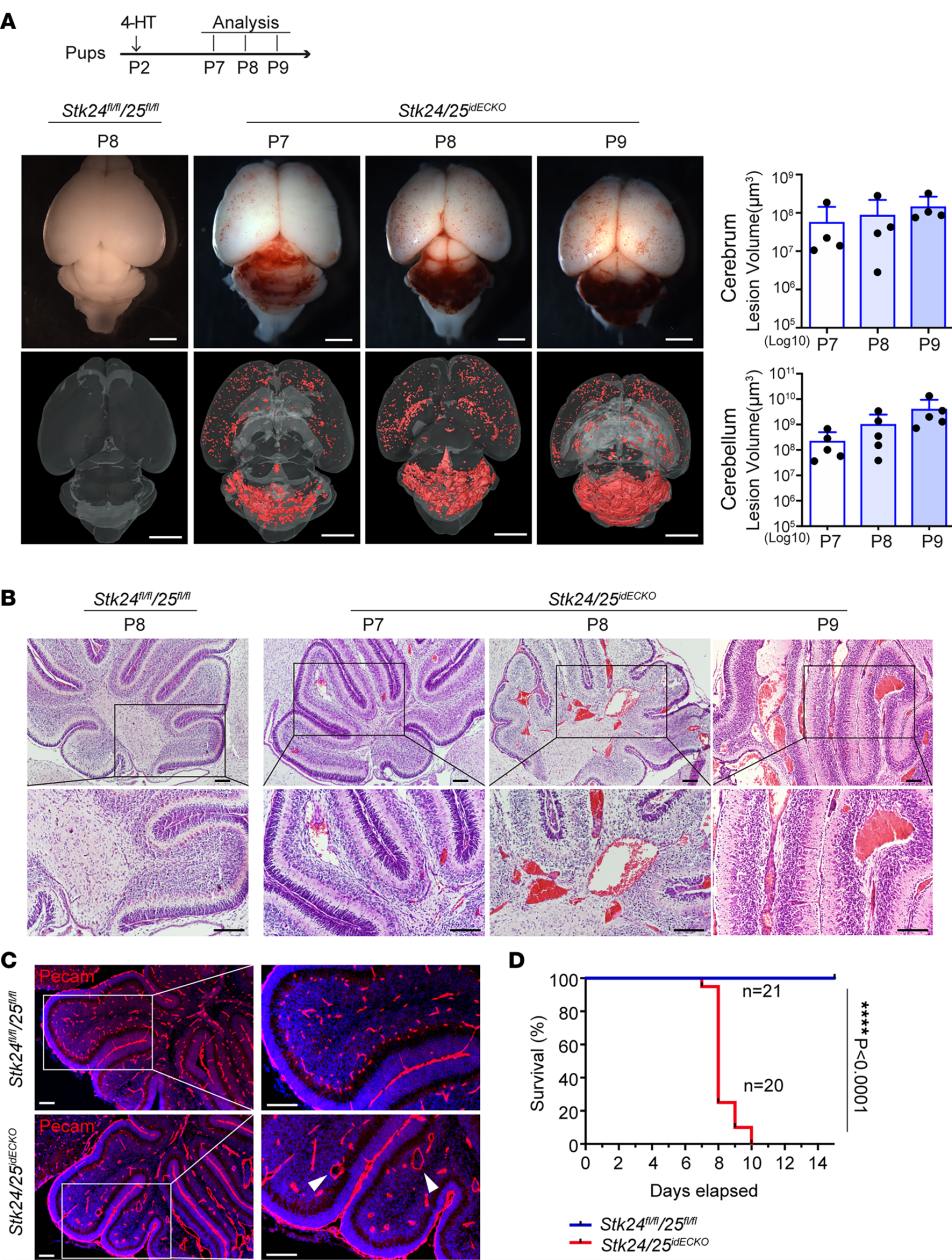
Development of cerebral cavernous malformations in the *Stk24/25^idECKO^* mice. (**A**) Schematic of 4-hydroxytamoxifen (HT) injection and sample collection. Pups were intragastrically injected with 4-HT at P2, and the brain tissues were harvested at specific time points (P7, P8, and P9). Stereomicroscopic images and μCT images of CCM lesions in control and the *Stk24/25^idECKO^* mice at P7, P8, and P9. Scale bars: 2 mm. Quantitative analysis of lesion volume in the cerebrum (*n* = 4) and cerebellum (*n* = 5) in the *Stk24/25^idECKO^* mice is shown on the right. Data in the quantitative plots are presented as mean ± SD. (**B**) H&E staining of brain sections in *Stk24^fl/fl^Stk25^fl/fl^* mice (*n* = 3) at P8 and *Stk24/25^idECKO^* mice (*n* = 3) at P7, P8, and P9. CCM lesions are shown as red masses. Scale bars: 100 μm. (**C**) Immunostainings of Pecam show cavernomas and reduced vascular number in *Stk24/25^idECKO^* mice (*n* = 4) compared with *Stk24^fl/fl^;Stk25^fl/fl^* mice (*n* = 4). The white arrowheads indicate malformed vessels. Scale bars: 50 μm. (**D**) The survival curve of *Stk24/25^idECKO^* (*n* = 20) and *Stk24^fl/fl^;Stk25^fl/fl^* (*n* = 21) mice after 4-HT induction at P2. Statistical analysis was performed using the Mantel-Cox test. ****P < 0.0001. Representative images from at least 3 or more independent experiments are shown.

We next determined whether the loss of *Stk24/25* could also cause CCM as previous reported in *Ccm* gene deficient mice (18, 19). We again induced *Stk24/25* gene deletion in *Stk24/25^idECKO^* mice by intragastric injection of 4-HT at P2 (Figure 3A). Robust CCM lesions were detected in both the cerebrum and the cerebellum of the *Stk24/25^idECKO^* mice starting from 5 days after 4-HT induction as determined by μCT (Figure 3A and Supplemental Figure 7) and histology (Figure 3, B and C). Malformed cavernous vessels were only detected in the brain and retina and were not found in other peripheral organs such as the lung, liver, or testes (Supplemental Figure 8). Administration of 4-HT to *Stk24/25^idECKO^* mice at P2 only allowed survival of the pups up to P10 (Figure 3D). The few *Stk24/25^idECKO^* mice that survived up to P10 displayed severe hemorrhage in the cerebellum (Figure 3A). Our data show that a loss of *Stk24/25* in endothelial cells caused a more severe phenotype than in previously reported models of inducible endothelial deletion of *Ccm1/2/3* by *Cdh5-CreERT2* (10, 18, 20).

**Figure 4:**
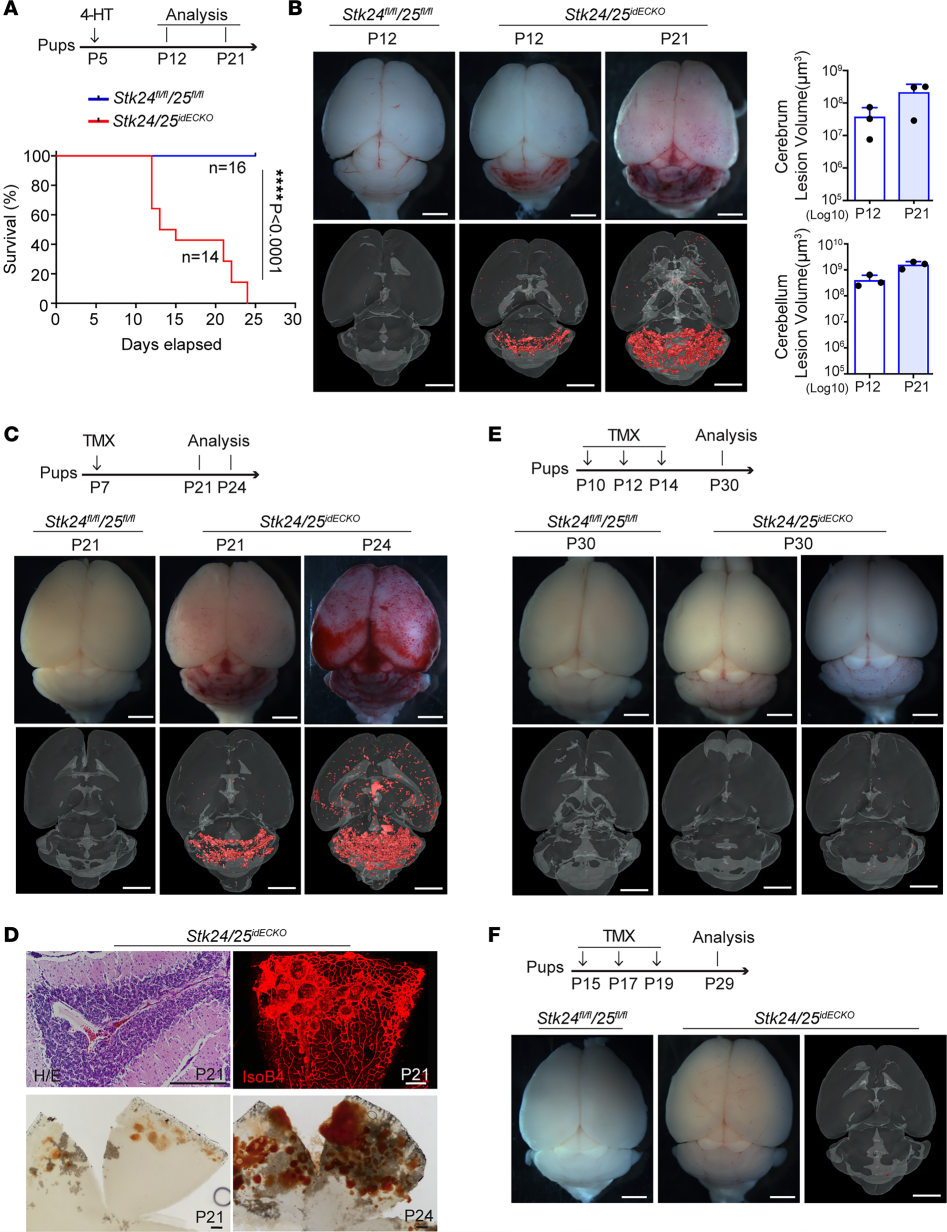
Limited induction time window of CCM formation in the *Stk24*- and *Stk25*-deficient mice. (**A**) Schematic of 4-HT injection and sample collection. Pups were intragastrically injected with 4-HT at P5, and the brain tissues were harvested at P12 and P21. The survival curves of *Stk24/25^idECKO^* (*n* = 14) and *Stk24^fl/fl^;Stk25^fl/fl^* mice (*n* = 16) after 4-HT induction at P5 are shown below. Statistical analysis was performed using the Mantel-Cox test. ****P < 0.0001. (**B**) Stereomicroscopic images and μCT images of CCM lesions in *Stk24/25^idECKO^* mice at P12 and P21 with 4-HT induction at P5. Scale bars: 2 mm. Quantitative analysis of lesion volume in cerebrum (*n* = 3) and cerebellum (*n* = 3) at P12 and P21 is shown on the right. Data are presented as mean ± SD. (**C**) Stereomicroscopic images and μCT imaging of CCM lesions in *Stk24/25^idECKO^* (*n* = 3) mice after tamoxifen induction at P7. Scale bars: 2 mm. (**D**) H&E staining of brain sections and whole-mount images showing CCM in brain and retina of *Stk24^fl/fl^;Stk25^fl/fl^* (*n* = 3) and *Stk24/25^idECKO^* mice (*n* = 3) at different time points. Scale bars: 200 μm. (**E**) Stereomicroscopic images and μCT images showing diminished CCM lesion formation in the *Stk24/25^idECKO^* mice (*n* = 3) at P30 after tamoxifen induction starting at P10. Scale bars: 2 mm. (**F**) Stereomicroscopic images and μCT imaging showing near absence of CCM lesion formation in the *Stk24/25^idECKO^* mice (*n* = 3) at P29 after tamoxifen induction starting at P15. Scale bars: 2 mm. Representative images from 3 or more independent experiments are shown.

The induction window of CCM lesion formation is limited to the first week of postnatal life when using established models of endothelial deletion of *Ccm1/2/3* genes with *Cdh5-CreERT2*. Since the *Stk24/25^idECKO^* mice showed such a severe CCM lesion burden, we investigated whether a delayed deletion of *Stk24/25* expression would still lead to the formation of the CCM lesions. When 4-HT was administered at P5, about 40% the *Stk24/25^idECKO^* mice survive up to P23 (Figure 4A) and robust CCM lesions were detected in brains at P12 and P21 (Figure 4B and Supplemental Figure 9A). In comparison with those mice induced at P2, the CCM lesion burdens in the P5 induced *Stk24/25^idECKO^* mutants were less severe in both the cerebrum and cerebellum (Figure 4B). When induced at P7, CCM lesions also developed in the brain and retina of *Stk24/25^idECKO^* mice at P21 and P24 (Figure 4, C and D). However, *Stk24/25* gene deletion at P10 and P15 resulted in no detectable CCM lesion when imaged up to P30 (Figure 4, E and F, and Supplemental Figure 9, B and C). These results indicate that the induction window of *Cdh5-CreERT2*–driven *Stk24/25* deletion was also limited to the first weeks after birth, similar to that of the *Cdh5-CreERT2*–driven *Ccm1/2/3* mutant mice, with more robust lesion formation with *Stk24/25* loss.

### STK24/25 function upstream of MEKK3-KLF2/4 signaling.

**Figure 5:**
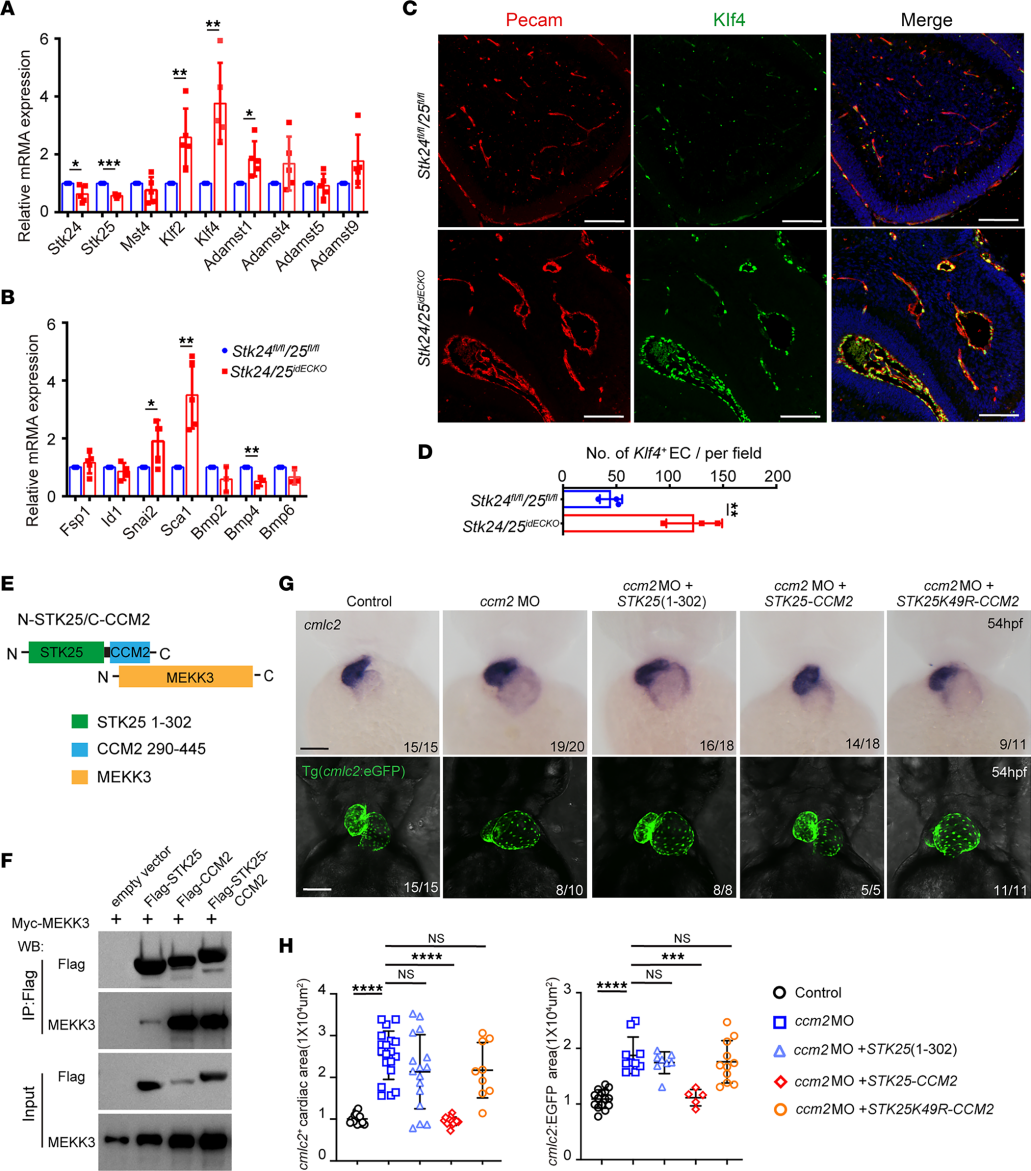
*Stk24* and *Stk25* deletion induces CCMs via MEKK3-KLF2/4 signaling activation. (**A** and **B**) Relative mRNA expression level of CCM-related genes in endothelial cells isolated from control and *Stk24/25^idECKO^* mice at P6 after induction at P2. *n* = 5 for each group — except Bmp2, Bmp4, and Bmp6, for which *n* = 3 was used. (**C**) Pecam and Klf4 immunofluorescence staining in endothelial cells of *Stk24^fl/fl^;Stk25^fl/fl^* (*n* = 3) and *Stk24/25^idECKO^* mouse brains (*n* = 3) at P6 after induction at P2. Scale bars: 100 μm. (**D**) Quantitative analysis showing increased *Klf4*^+^ EC in *Stk24/25^idECKO^* mice compared with *Stk24^fl/fl^;Stk25^fl/fl^* mice. The quantitative data (mean ± SD) from 3 independent experiments are reported, and significance was determined using unpaired *t* test. **P < 0.01. (**E**) Schematic representation of the interaction between MEKK3 and STK25-CCM2 hybrid protein consists of N terminal kinase domain of STK and C terminal MEKK3 interacting domain of CCM2. (**F**) Immunoprecipitation experiment shows that STK25/CCM2 hybrid protein interaction with MEKK3 was comparable with that of CCM2. (**G**) Representative images of in situ staining of *cmlc2* and fluorescence imaging of the hearts of Tg (*cmlc2*:EGFP) zebrafish embryos in which myocardial cells express EGFP. The *ccm2* morpholino induced dilated heart, while coinjection with mRNA expressing STK25-CCM2 rescued the dilated heart phenotype compared with injection of mRNA only expressing STK25(1-302) or STK25K49R-CCM2 hybrid protein. Scale bars: 100 μm. (**H**) Quantification of *cmlc2*^+^ cardiac area and *cmlc2*:EGFP area of zebrafish embryos with *ccm2* morpholino and different cRNA. Data are presented as mean ± SD, and significance was determined using unpaired *t* test (**A**, **B**, and **D**) or 1-way ANOVA (**H**). *P < 0.05, **P < 0.01, ***P < 0.001, ****P < 0.0001.

MEKK3-KLF2/4 and bone morphogenetic protein (BMP) signaling has been reported to function downstream of CCM signaling for CCM lesion formation (10, 20, 21). As a first step, we analyzed the relative mRNA expression levels of genes encoding various components of these signaling pathways by quantitative PCR (qPCR). We treated P2 control and *Stk24/25^idECKO^* mice with 4-HT and isolated P6 endothelial cells from these mice. The *Stk24/25^idECKO^* endothelial cells demonstrated significantly enhanced MEKK3 signaling, as shown by the increased expression of downstream effectors, *Klf2/4* and *Adamts1,* over littermate controls (Figure 5A). Among the genes implicated in BMP and endothelial-mesenchymal transition (EndoMT) signaling, the expression of *Snai2* and *Sca1* were also significantly increased, while *Bmp4* expression was decreased (Figure 5B). The upregulation of *Klf4* expression was also evident at the protein level, shown by the increased immunostaining in endothelial cells (Figure 5, C and D).

CCM3 interacts with STK24/25, and CCM2 interacts with MEKK3, while the interaction between CCM2 and CCM3 is essential to prevent CCM lesion formation (2, 4, 5, 9). However, it is unknown whether a close proximity between STK24/25 and MEKK3 is required for the suppression of MEKK3 activation. To test this possibility, we generated a hybrid protein consisting of the N-terminal kinase domain of STK25 and the C-terminal domain of CCM2, which mediates the interaction between CCM2 and MEKK3, as schematically shown in Figure 5E. The predicted interaction between the STK25-CCM2 fusion protein and MEKK3 was experimentally confirmed by co-IP experiments (Figure 5F). We then expressed this hybrid protein in a ccm2 morphant zebrafish, which led to a dilated heart phenotype. The expression of STK25-CCM2, but not the N-terminal kinase domain of STK25 (STK25[1-302]) or the STK25-CCM2 kinase-dead hybrid protein (STK25K49R-CCM2) in the ccm2 morphant, was able to reverse the dilated heart phenotype (Figure 5, G and H). These experiments suggest that proximity between STK and MEKK3 is necessary and sufficient for the suppression of MEKK3 activity in the zebrafish model.

## Discussion

In this study, we found that loss of STK24/25 impairs angiogenesis and causes CCM lesion formation, which is consistent with recent work that was published while this manuscript was in preparation (22). In addition, we found that the proximity between STK24 and MEKK3 is sufficient to rescue the dilated heart phenotype in zebrafish. Previous work in zebrafish had identified STK24/25 as components of the CCM signaling pathway. Decreasing gene dosage of *ccm3* and *stk24/25* resulted in a typical dilated heart phenotype, similar to the phenotype observed in *ccm* mutant zebrafish (4). In this study, we did not find vascular phenotypes in mice lacking 3 out of 4 alleles of *Stk24* and *Stk25* genes in endothelial cells. However, complete deletion of both Stk24 and Stk25 in endothelial cells resulted in a phenotype that was identical to that of the *Ccm1-* or *Ccm2*-deficient mouse embryos. Together, these data suggest that (a) STK24 and STK25 play redundant roles in the CCM pathway regulating vascular development and (b) STKs are bona fide members of the CCM pathway.

Like Ccm1 and Ccm2, Stk24/25 are also required for lumen formation of the BAA and DA. In angiogenesis, STK24/25 appear to have a role in limiting filopodia in tip cells and in promoting the elongation and remodeling of vascular plexus. Interestingly, the absence of STK24/25 had no effect on the remodeling of the arterial branch of the vascular network, suggesting the possibility that the STK24/25-mediated remodeling mechanism can be overwritten by oxygen tension or arterial signaling such as a Notch-driven remodeling process. In mouse brain, endothelial deficiency of Stk24/25 causes the disorganization of vascular networks and the formation of dilated vascular caverns. This may be due to similar mechanisms that cause remodeling defects in retinal vessels. Whether these processes involve stress fibers or ERM protein regulation downstream of CCM is not clear.

Deletion of Stk24/25 causes restriction of BAA and DA as well as dilation of the microvasculature of the developing brains or postnatal brains. This suggests that the phenotypic presentation of CCM signal deficiency in endothelial cells is likely dependent on tissue context or endothelial cell identity. It is not known whether the restriction of major arteries and the dilation of brain microvasculature have a similar underlying endothelial cell mechanism. It is possible that the cytoskeletal changes conferred by CCM signaling deficiency in endothelial cells increase cellular motility. In venous endothelial cells of the microvasculature, deletion of CCM signaling flattens the endothelial cells and causes dilation of microvasculature, whereas in arterial endothelial cells, loss of CCM signaling may drive these cells to migrate inward and restrict lumen formation. A careful analysis of endothelial cell behavior in vivo and the differential gene expression profile changes between arterial and venous endothelial cells may shed light on why CCMs only occur in the venous vessels of neuronal tissue.

As expected from zebrafish studies, deletion of STK24/25 in endothelial cells in postnatal mice causes CCM lesions. However, the phenotype in the *Stk24/25^idECKO^* mice was more severe when compared with *Ccm1* or *Ccm2* deletion using the same *Cdh5-CreERT2* line (23). Robust CCM lesions formed in both the cerebrum and the cerebellum upon deletion of *Stk24/25* early after birth. The aggravated phenotype might be due to a higher potency of Stk24/25 to affect downstream signals involved in cell morphology, resulting in a more severe CCM phenotype. Another difference between mice with Stk24/25 and Ccm1/2/3 endothelial cell deletion was that the formation of CCM lesions by Stk24/25 deletion can occur over a broader time window, as the formation of lesions occurred even when tamoxifen was administered at P7. In the *Ccm2*-deficient mice, very few lesions can be induced at this time point (10, 18). The broader and robust induction window makes the lesion burden in the induced mice less variable and better suitable for intervention studies.

The downstream signaling analysis indicates that *Stk24/25* deficiency affects the MEKK3-KLF2/4 pathway. In addition, transcriptional upregulation was also observed for *Sca1*, one of the genes previously reported in BMP/TGF-β–mediated EndoMT process (21). The function of this gene in CCM pathogenesis remain to be studied.

This study also revealed that Stk24/25 ablation leads to increased *Klf2/4* expression, which in turn may contribute to EndoMT signaling. It is currently unknown whether this process also involves MEKK3-derived signals. Our hybrid protein experiments suggest that proximity of the STK kinase domain to MEKK3 is sufficient to bypass the requirement of CCM2 to transduce signal and suppress MEKK3 activity. It is possible that STK24/25 could directly regulate MEKK3 activity through protein phosphorylation that inhibits its function. Indeed, phosphorylation of MEKK3 at Tyr294 has been shown to prevent MEKK3 activity that contributes to activation of NF-κB (24). Alternatively, the association of STK24/25 with MEKK3 might mask an interaction surface or lead to structural changes that inactivate its kinase function. More mechanistic insight is required in order to develop allosteric compounds that interfere with MEKK3 activation when CCM proteins are missing. Possible mode of action of these compounds could be based on the induced interaction between STK24/25 and MEKK3 or on the induction of inhibitory MEKK3 phosphorylation.

## Methods

### Mice.

*Cdh5-CreERT2*, *Stk24^fl/fl^* and *Stk25^fl/fl^* mice have been described previously (23, 25). *Tie2-Cre* mice were purchased from The Jackson Laboratory. Experimental animals were maintained on a C57BL/6J;129 mixed genetic background.

### Mouse embryo processing.

For whole-mount staining, embryos were fixed in 4% (v/v) PFA at 4°C overnight. After fixing embryos were washed with PBS and dehydrated and rehydrated in graded methanol series. The samples were blocked in PBS containing 0.5% (v/v) TritonX-100 (PBST) and 2% (w/v) milk powder at room temperature for 2 hours. The embryos were incubated with the primary antibody endoglin (rat anti-endoglin, MAB1320-SP, Bio-Techne, 1:200 dilution) at 4°C overnight with rocking, followed by washing 3 times in 2% milk/PBST solution. Embryos were then incubated with goat anti–rat IgG cross-adsorbed secondary antibodies (Alexa Fluor 594–conjugated, A11007, Invitrogen, 1:1,000 dilution) at 4°C overnight with rocking. After washing, embryos were embedded in fluorescence mounting medium (S3023, Dako) and imaged with a Zeiss Axio-Imager LSM-800 confocal microscope (Carl Zeiss).

To examine the development of BAA and DA, embryos were injected with India ink through a glass pipette inserted into the left ventricle of contracting hearts. Then, embryos were fixed with 4% PFA for 3 minutes after ink injection and were imaged under a stereomicroscope (Olympus SZX16).

### Induction of Stk24/25 deletion in vivo.

For the in vivo induction of *Stk24/25* gene deletion in neonatal *Cdh5-CreERT2;Stk24^fl/fl^;Stk25^fl/fl^* mice, 4-HT (Sigma-Aldrich; H7904) and tamoxifen were dissolved in corn oil at the concentration of 0.5 mg/mL and 20 mg/mL, respectively. The pups were intragastrically injected with a single dose (75 μL, 37.5 μg) 4-HT at P2 or P5. For induction of P7 mice, tamoxifen was fed at a single dose of (200 μL, 4 mg). For inductions starting at P10 or P15, tamoxifen (250 μL, 5 mg) was delivered by gavage a total of 3 times every other day.

### Contrast-enhanced, x-ray μCT.

Postnatal pups were anesthetized with Avertin (Sigma-Aldrich; T48402) and underwent intracardiac perfusion with PBS and 2% paraformaldehyde (w/v). Brains were immediately placed in 4% PFA/PBS fixative. Brains remained in fixative until staining with Lugol’s solution (Sigma-Aldrich; L6146) for 48 hours and were subjected to μCT imaging. Brains were randomized and scanned by blinded operators using an Xradia Micro-CT system (Xradia MicroXCT-400, Xradia). Images were acquired at 50 kV, 10 W, 721 projections, and 3-second integration per 180° rotation.

For postlesion labeling, the brain image stacks were volume rendered and overlaced with the labeled lesions in the Avizo 3D environment. All tissue processing, imaging, and volume quantification were done in a blinded manner by investigators at Tianjin Medical University without any knowledge of experimental details.

### Brain histological analysis.

Brains harvested from mice were fixed with 4% PFA and embedded in paraffin. Paraffin sections were stained for H&E staining using standard protocols. For Pecam immunostaining, deparaffinized sections (7 μm) were subjected to rehydration, followed by antigen retrieval by heating in citrate buffer (Beyotime, P0083) for 20 minutes. Endogenous peroxidase was blocked with 3% hydrogen peroxide for 15 minutes. After blocking in a PBST solution containing 10% donkey serum (Jackson ImmunoResearch) and 1% BSA for 1 hour, the sections were incubated with primary antibody (rat anti-Pecam, Dianova DIA-310, Dianova, 1:300 dilution) overnight at 4°C. After washing, the slides were incubated with undiluted ImmPRESS (Peroxidase) secondary antibody (goat anti-rat, Vector Laboratories, MP-7444) for 60 minutes at room temperature, washed with PBST, and then incubated with TSA Fluorescence System Working solution (TSA-plus tetramethylrhodamine System, NEL742001KT, PerkinElmer) at room temperature for 8 minutes. The sections were washed with PBST and mounted with mounting solution containing DAPI (Vector Laboratories). For Klf4 immunostaining, the sections were incubated with primary antibody (goat anti-Klf4 antibody, AF3158, R&D, 1:200 dilution) overnight at 4°C. After washing, the slides were incubated with donkey anti–goat IgG (H+L) cross-adsorbed secondary antibody (Alexa Fluor 488 conjugate, A11055, Invitrogen, 1:500 dilution). The sections were washed with PBST and mounted with mounting solution containing DAPI (Vector Laboratories).

For the analysis of vasculature development, the brain samples were fixed in 4% PFA and incubated at 4°C for 2 hours. Subsequently samples were embedded in 3% low–melting point agarose, and coronal vibratome sections (100 μm) were made. The vibratome sections were permeabilized in PBS solution containing 0.3% TritonX-100 and 2% BSA for 2 hours at room temperature. After PBS washing, the sections were incubated with isolectin-B4 (DL1207, Vector, 1:200 dilution) for 2 hours. Imaging was performed using a Zeiss Axio-Imager LSM-800 confocal microscope and a Nikon microscope (Eclipse Ni).

### Fluorescence staining of whole-mount retinas.

Eyes were collected from neonatal mice on P6, P15, or P60 and then fixed in 4% PFA for 2 hours at room temperature. Retinas were isolated and permeabilized in 0.3% TritonX-100 and 1% BSA/PBS overnight at 4°C. Followed by incubation with Isolectin B4 or primary antibody in 0.3% TritonX-100 and 5% donkey serum in 5% BSA/PBS overnight at 4°C. For Erg1 staining, the retinas were washed several times in PBS and incubated with fluorescent secondary antibodies. Reagents used were as follows: Dylight 594 isolectin-B4 (DL1207, Vector, 1:200 dilution), rabbit anti-ERG (ab92513, Abcam, 1:200 dilution), IgG (H+L) cross-adsorbed donkey anti–rabbit DyLight 488 (SA5-10038, Invitrogen, 1:500 dilution), phospho-Histone H3 (Ser10) mouse mAb (9706, CST, 1:200 dilution), and Alexa Fluor 647 conjugate (4410, CST, 1:500 dilution). Imaging was performed using a Zeiss Axio-Imager LSM-800 confocal microscope.

### Isolation of cerebellar endothelial cells and qPCR analysis.

Cerebellar endothelial cells were isolated from mice by enzymatic digestion, followed by separation using MACS by anti-CD31–conjugated magnetic beads (MACS MS system, Miltenyi Biotec). Mice were first anesthetized with Avertin (Sigma-Aldrich) and perfused with sterile PBS. Cerebellums of the mice were digested with 1 mg/mL collagenase/dispase solution (Roche, 10269638001) and bezonase (MilliporeSigma, E1014, 1:2,000 dilution) in complete DMEM for 10 minutes at 37°C with gentle shaking. The digestion was then passed through a 70 μm cell strainer (BD Biosciences). Cells were centrifuged (350*g* for 5 minutes at 4°C), resuspended, and incubated with anti–mouse CD31 antibody–conjugated microbeads (Miltenyi Biotec, 130-097-418) for 15 minutes at 4°C. Microbead-bound cells were then washed and separated using MACS MS columns according to the vendor′s protocol. Cells bound to the magnetic column were eluted and centrifuged (700*g* for10 minutes at 4°C) for qPCR analysis.

The total RNA was extracted using TRIzol Reagent (Thermo Fisher Scientific, 15596018), and complementary DNA (cDNA) was synthesized using StarScript II First-strand cDNA Synthesis Kit (GenStar, A212-10). Real-time PCR was performed with the ChamQ Universal SYBR qPCR Master Mix (Vazyme Biotech Co., Q711-02/03). The following primers were used in this study: *Gapdh* forward: 5′- GTCCCGTAGACAAAATGGTGA -3′; *Gapdh* reverse: 5′- TTTGATGTTAGTGGGGTCTCG -3′; *Stk24* forward: 5′- CAGCTGACGGATACCCAGATC -3′; *Stk24* reverse: 5′- GTAGTTCCCTTCCAGTGTGGG -3′; *Stk25* forward: 5′- CTGCACTGGACTTGCTGAAAC -3′; *Stk25* reverse: 5′- GGACCAGATGTCAGCCTTGAA -3′; *Mst4* forward: 5′- CATTGGATCTTCTGCGTGCTG -3′; *Mst4* reverse: 5′- CCAAAACGGAGTCCCTACGAA -3′; *Klf2* forward: 5′- CGCCTCGGGTTCATTTC -3′; *Klf2* reverse: 5′- AGCCTATCTTGCCGTCCTTT -3′; *Klf4* forward: 5′- GTGCCCCGACTAACCGTTG -3′; *Klf4* reverse: 5′- GTCGTTGAACTCCTCGGTCT -3′; *Adamts1* forward: 5′- CCTTACGGCAGCAGACACA -3′; *Adamts1* reverse: 5′- AATCTGCTGTCAGTGGCCC -3′; *Adamts4* forward: 5′-CAGTGCCCGATTCATCACT-3′; *Adamts4* reverse: 5′-GAGTCAGGACCGAAGGTCAG -3′; *Adamts5* forward: 5′-CGACCCTCAAGAACTTTTGC-3′; *Adamts5* reverse: 5′-CGTCATGAGAAAGGCCAAGT-3′; *Adamts9* forward: 5′- AGCGGAAAATCAGAATGCGAAAA-3′; *Adamts9* reverse: 5′- TGAAGGTTTGCTCCGTGGTATAA-3′; *Fsp1* forward: 5′- TGGTCTGGTCTCAACGGTTAC -3′; *Fsp1* reverse: 5′- ACTTCATTGTCCCTGTTGCTG -3′; *Id1* forward: 5′- ATCCTGCAGCATGTAATCGAC-3′; *Id1* reverse: 5′- GAGTCCATCTGGTCCCTCAGT-3′; *Snai2* forward: 5′- TATGGACATCGTCGGCAGC -3′; *Snai2* reverse: 5′- GCAGATGTGCCCTCAGGTT -3′; *Sca1* forward: 5′- TGTGCAGAAAGAGCTCAGGG -3′; *Sca1* reverse: 5′- TCAGGCTGAACAGAAGCACC -3′; *Bmp2* forward: 5′- AGATCTGTACCGCAGGCACT -3′; *Bmp2* reverse: 5′- GTTCCTCCACGGCTTCTTC-3′; *Bmp4* forward: 5′- CTGTGAGGAGTTTCCATCACGA -3′; *Bmp4* reverse: 5′- ATTTCTGCTGGGGGCTTCATAA-3′; *Bmp6* forward: 5′- CGACAAGGAGTTCTCCCCAC -3′; and *Bmp6* reverse: 5′- AGCCAACCTTCTTCTGAGGC -3′.

### Co-IP analysis.

Cultured HEK293 cells (ATCC, CRL-1573) were transfected with empty pcDNA5 vector or plasmids expressing Myc-MEKK3, Flag-STK25, Flag-CCM2, and Flag-tagged N-STK25(1-302)/C-CCM2(290-445). After 48 hours of transfection, cells were lysed with immunoprecipitation assay buffer and subjected to pulldown with anti-Flag antibody affinity gel (Sigma-Aldrich). After washing, proteins bound to the beads were eluted and subjected to Western blot analysis.

### Zebrafish studies.

Tg (*cmlc2:*EGFP) zebrafish were obtained from the Zebrafish International Resource Center (ZIRC). Morpholino oligonucleotides were obtained from Gene Tools and were injected into the yolk of 1-cell-stage embryos at the dosage of 4 ng/embryo. To rescue the big heart phenotype conferred by *ccm2* morpholinos (13), 100 pg of mRNA encoding STK25-CCM2, STK25(1-302), or STK25-K49R-CCM2 was coinjected with the *ccm2* morpholino oligonucleotides. Zebrafish embryos were mounted in 2% methylcellulose. Fluorescence images of the heart were acquired using an Andor Dragonfly 505 confocal microscope (Oxford Instruments). For in situ hybridization, zebrafish embryos at indicated stage were fixed and probed with a cmlc2 probe; the images were acquired using a Nikon SMZ 1500 microscope equipped with a Nikon DXM1200F camera.

### Statistics.

The data in this study are expressed as the mean ± SD as noted in individual figure legends. Statistical analyses were performed using GraphPad PRISM software, version 9.0. The unpaired Student’s 2-tailed *t* test and 1-way ANOVA were used to assess the differences involving 2 and various groups, respectively. The Mantel-Cox test was used to assess the differences of survival curve. Differences were considered statistically significant when *P* < 0.05.

### Study approval.

The IACUC of Tianjin Medical University approved all animal ethics and protocols. All experiments were conducted under the guidelines/regulations of Tianjin Medical University, the guideline of National Research Council of the National Academies (26), and the Animal Research: Reporting of In Vivo Experiments (ARRIVE) guidelines.

## Author contributions

XY, STW, RG, and LW (Department of Hematology) designed and performed most of the experiments and wrote the manuscript. RW, YW, ZD, LW (Department of Pharmacology), and ZH performed experiments and analyzed data. CQ and XW analyzed data. RL and MLS analyzed data and wrote the manuscript. XZ designed and performed experiments and wrote the manuscript. All authors had access to the study data and reviewed and approved the final manuscript.

## Supplementary Material

Supplemental data

## Version 1 01/24/2023

In-Press Preview

## Version 2 03/08/2023

Electronic publication
