# Supplementary material for: Release of STK24/25 suppression on MEKK3 signaling in endothelial cells confers cerebral cavernous malformation
Source: JCI Insight. 2023 Mar 8;8(5):e160372. doi: 10.1172/jci.insight.160372 (PMC10077477; doi:10.1172/jci.insight.160372)
Supplement: Supplemental data [file jciinsight-8-160372-s006.pdf]

Supplementary Materials

Supplemental Table 1-2

Supplemental Figure 1-9

Supplemental Tables

Supplemental Table 1. Offspring of *Tie2Cre;Stk24*$^{fl/fl}$*25*$^{fl/+}$ x *Stk24*$^{fl/fl}$*Stk25*$^{fl/fl}$ matings at various developmental stages. No live *Tie2Cre;Stk24*$^{fl/fl}$*25*$^{fl/fl}$ embryos were observed after E11.

	**E9-E9.5**	**E10-E10.5**	**E11.5**	**P1**
Stk24$^{fl/fl}$*Stk25*$^{fl/+}$	26	27	10	6
Stk24$^{fl/fl}$*Stk25*$^{fl/fl}$	19	23	12	4
Tie2Cre;Stk24$^{fl/fl}$*Stk25*$^{fl/+}$	11	22	9	3
***Tie2Cre;Stk24*$^{fl/fl}$*Stk25*$^{fl/fl}$**	**19**	**22 (13 dead)**	**14 (dead)**	**0**
Total	75	94	45	13

Supplemental Table 2. Offspring of *Tie2Cre;Stk24*$^{fl/+}$*25*$^{fl/fl}$ x *Stk24*$^{fl/fl}$*Stk25*$^{fl/fl}$ matings at various developmental stages. No live *Tie2Cre;Stk24*$^{fl/fl}$*25*$^{fl/fl}$ embryos were observed after E11.

	**E9-E9.5**	**E11**	**E12-E12.5**	**E18.5**	**P1**
Stk24$^{fl/+}$*Stk25*$^{fl/fl}$	13	8	2	4	7
Stk24$^{fl/fl}$*Stk25*$^{fl/fl}$	12	7	5	5	9
Tie2Cre;Stk24$^{fl/+}$*Stk25*$^{fl/fl}$	16	6	5	3	5
***Tie2Cre;Stk24*$^{fl/fl}$*Stk25*$^{fl/fl}$**	**10**	**6 (dead)**	**0**	**0**	**0**
Total	51	27	12	12	21

Supplemental Figures

Supplemental Figure 1

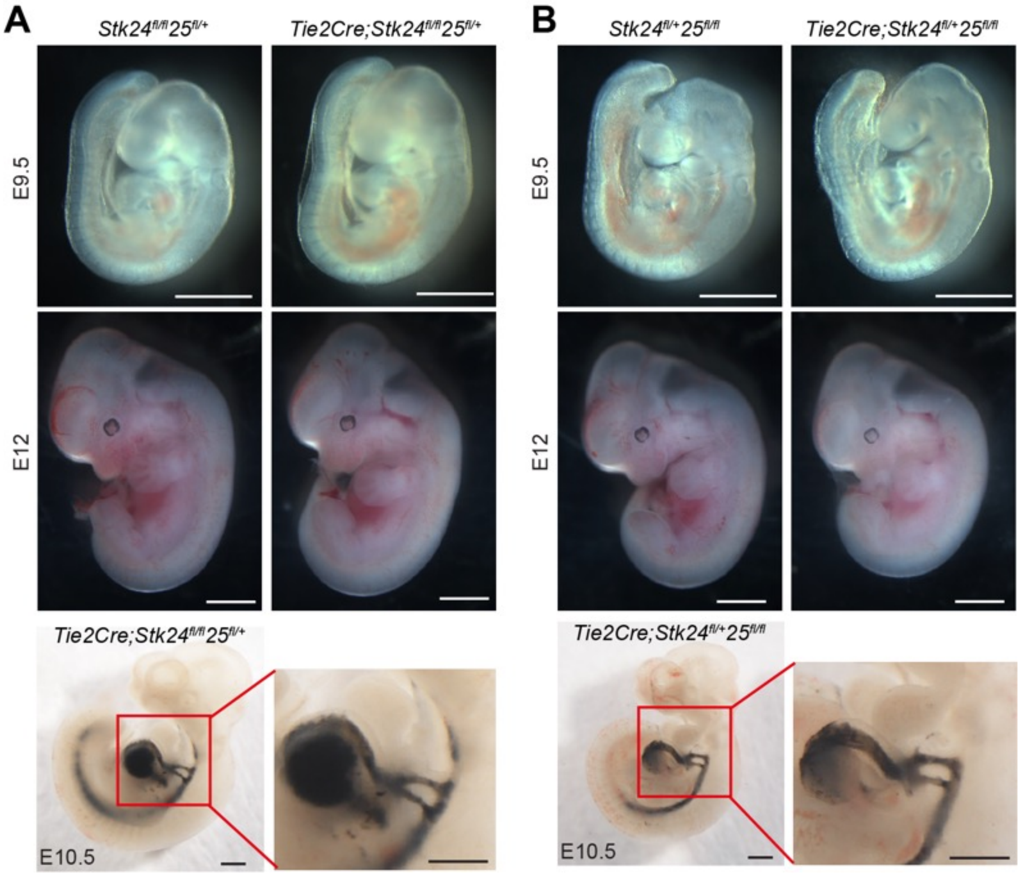

Fig. S1. Normal gross development of *Stk24* deficient or *Stk25* deficient embryos. A) Stereomicroscopic images of control and *Stk24* deficient embryos at E9.5 and E12. Scale bars, 1 mm. Indian ink injected hearts show normal patterning of BAA and DA. Scale bars, 500 µm. **B)** Stereomicroscopic images of control and *Stk25* deficient embryos at E9.5and E12. Scale bars, 1 mm. Indian ink injected hearts show normal patterning of BAA and DA (n=3 for *Stk24* deficient and *Stk25* deficient E10.5 embryos). Scale bars, 500µm.

Supplemental Figure 2

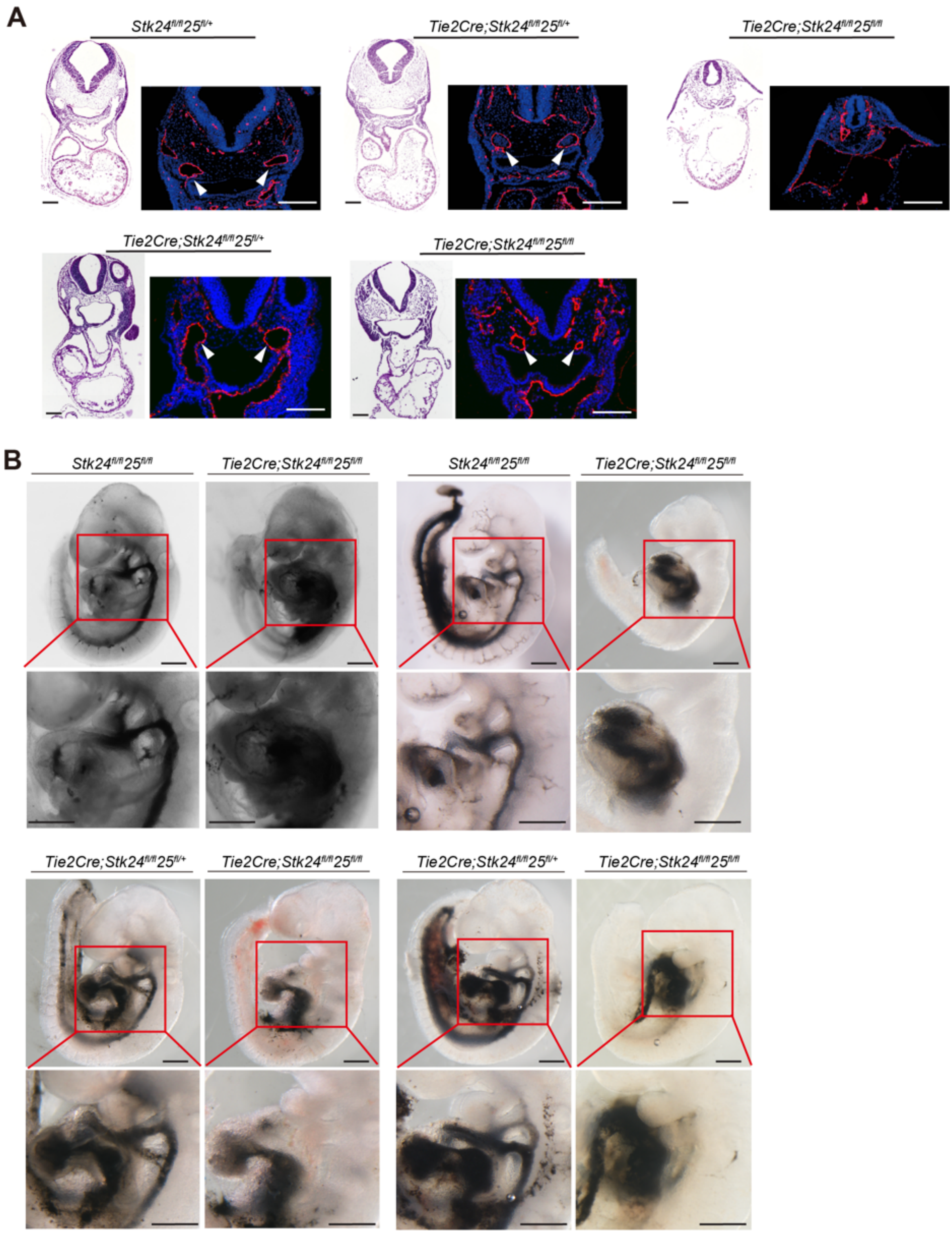

Fig. S2. Deletion of *Stk24* and *Stk25* in endothelium results in vascular defects during embryonic development. A) H&E staining and Pecam immunostaining of transverse sections of E10 embryos showing the presence of normally lumenized dorsal aortas (DA) in the control and $Tie2Cre;Stk24^{fl/fl}Stk^{25fl/+}$ embryos, but not in the $Stk24/25^{dECKO}$ embryos. White arrows indicate DA. Scale bars, 100 µm. **B**) Injection of Indian ink into the ventricle of embryonic hearts (E9.5) showing the establishment of a functional circulation in control and $Tie2Cre;Stk24^{fl/fl}Stk^{25fl/+}$ embryos that was inhibited in the $Stk24/25^{dECKO}$ embryos. Scale bars, 500 µm.

Supplemental Figure 3

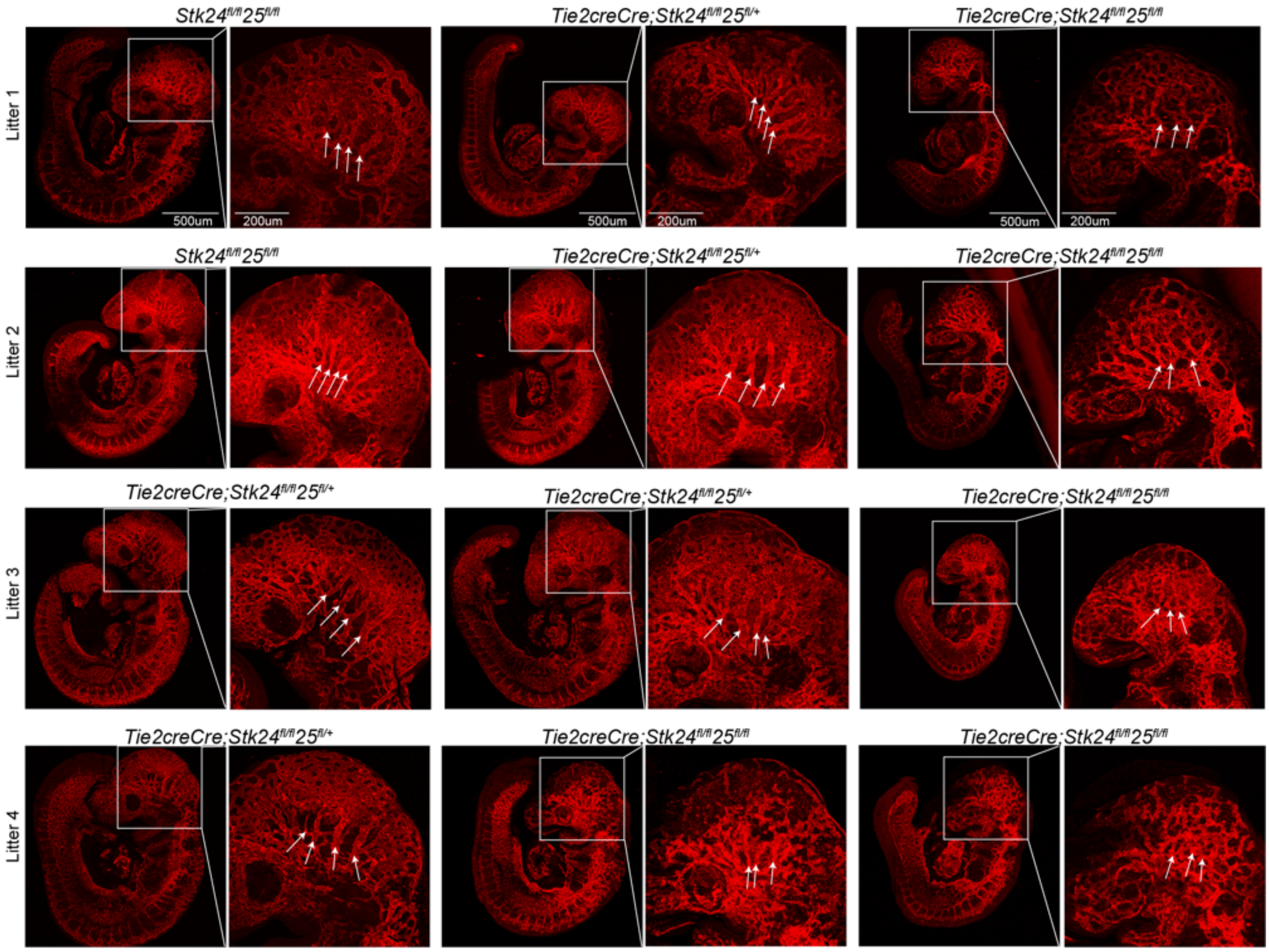

Fig. S3. Deletion of *Stk24* and *Stk25* in endothelium results in impaired vascular patterning during embryonic development. Whole-mount immunostainings of embryos with the endothelial marker endoglin in the brain of the E9.5 *Stk24/25*^{dECKO} embryos and its *Stk24*$^{fl/fl}$*Stk*$^{25fl/fl}$ and/or *Tie2Cre;Stk24*$^{fl/fl}$*Stk*$^{25fl/+}$ littermate controls. White arrows indicate regions of vascular patterning. Scale bars represent 500 µm in low magnification images, and 200 µm in enlarged images.

Supplemental Figure 4

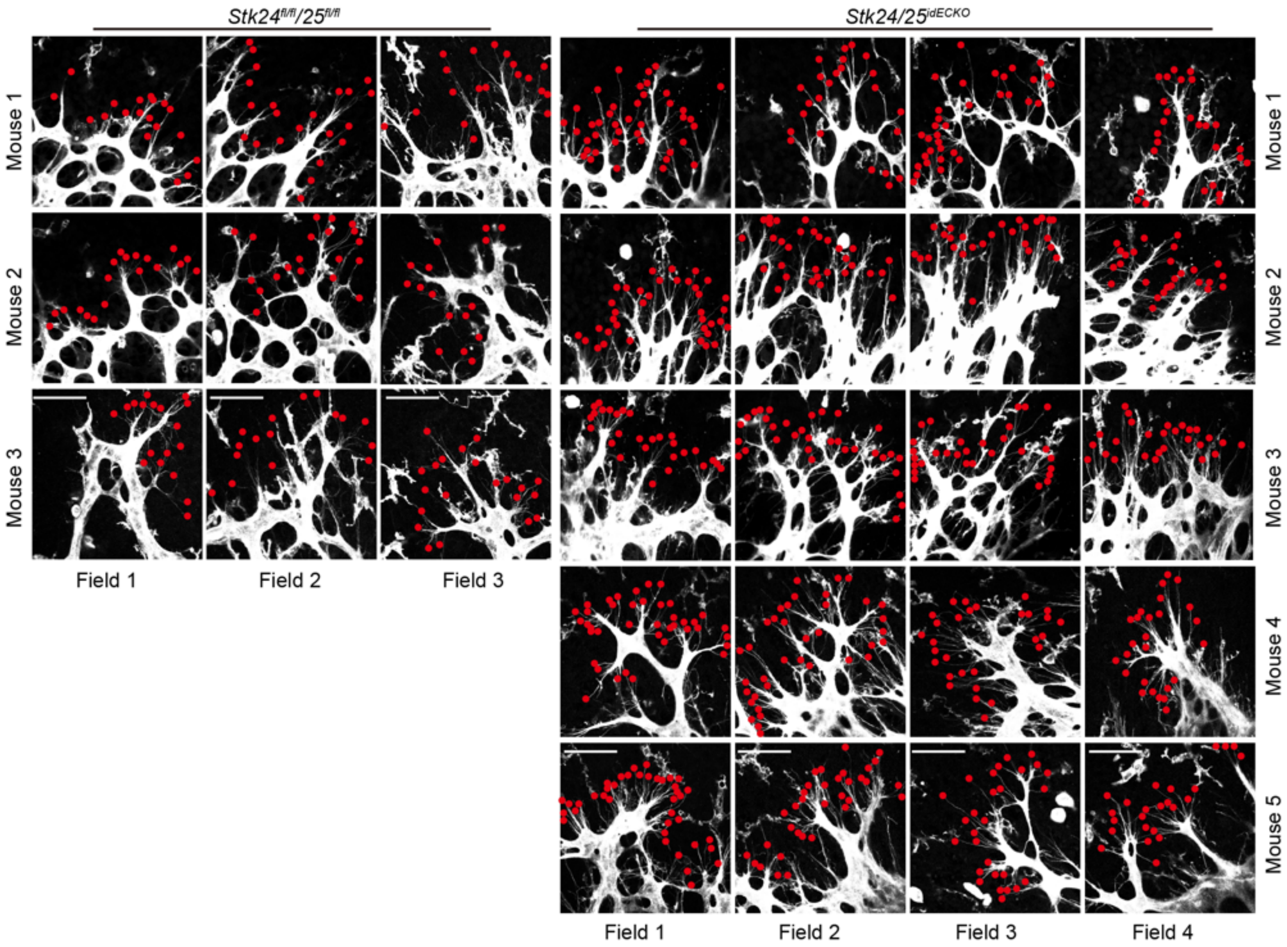

Fig.S4. Increased filopodia numbers in *Stk24* and *Stk25* deficient mice. Confocal images of P6 retinal lobes stained with IsoB4 in the *Stk24/25*^{idECKO} mice (n=5) compared to the *Stk24*$^{fl/fl}$*Stk25*$^{fl/fl}$ mice (n=3). The red dots denote filopodia in the vascular front. Scale bars, 100 µm. Three fields were quantified for each control retina while four fields were quantified for each *Stk24/25*^{idECKO} mouse.

Supplemental Figure 5

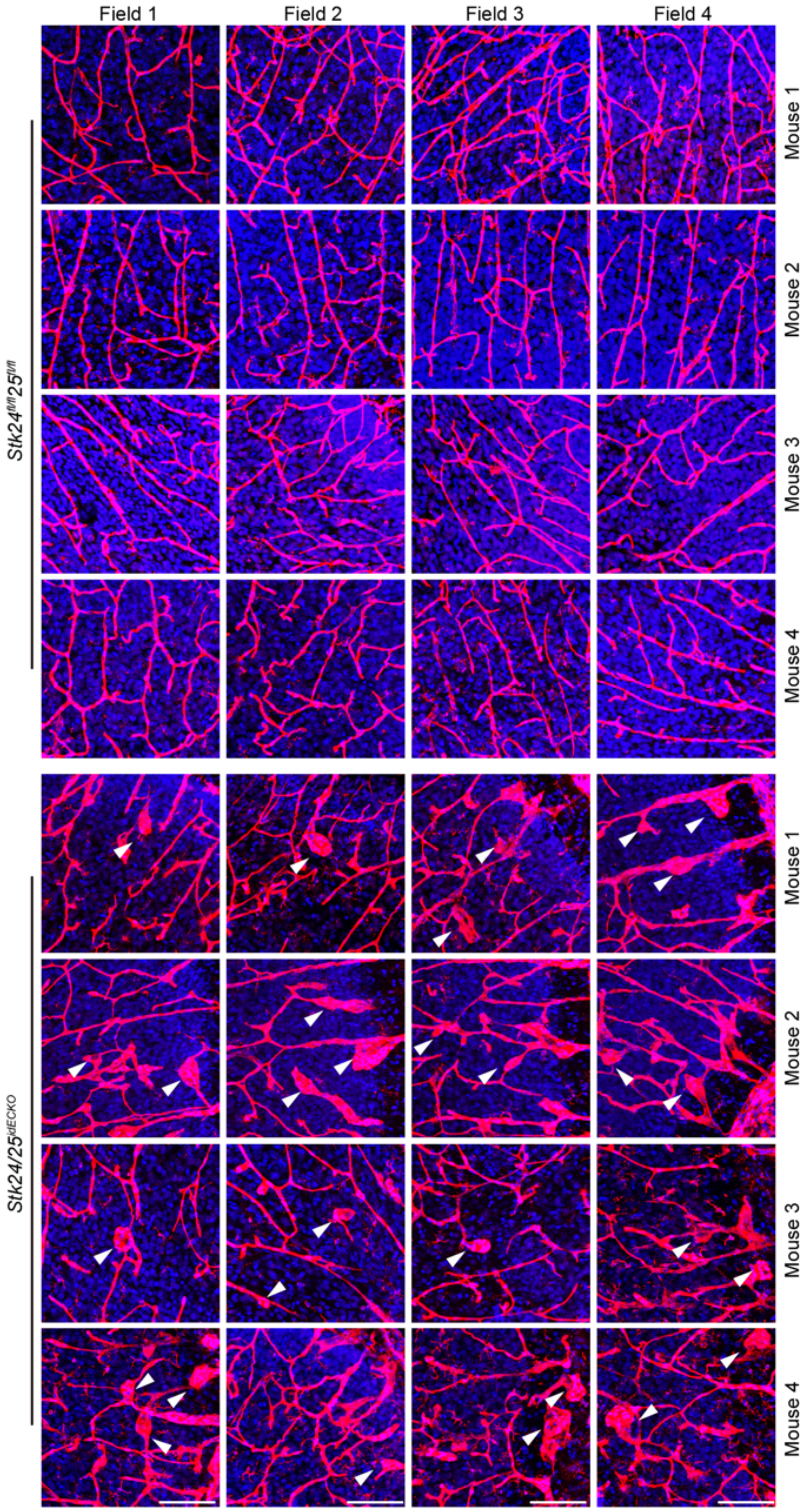

Fig. S5. Deletion of *Stk24* and *Stk25* in endothelium of new born pups resulted in malformed vessels in the cerebrum. Confocal images of IsoB4 staining in the cerebral cortical vasculature of P8 control and *Stk24/25*^{idECKO} pups. The white arrows indicate the malformated cavernous vessels. Scale bars, 100 µm.

Supplemental Figure 6

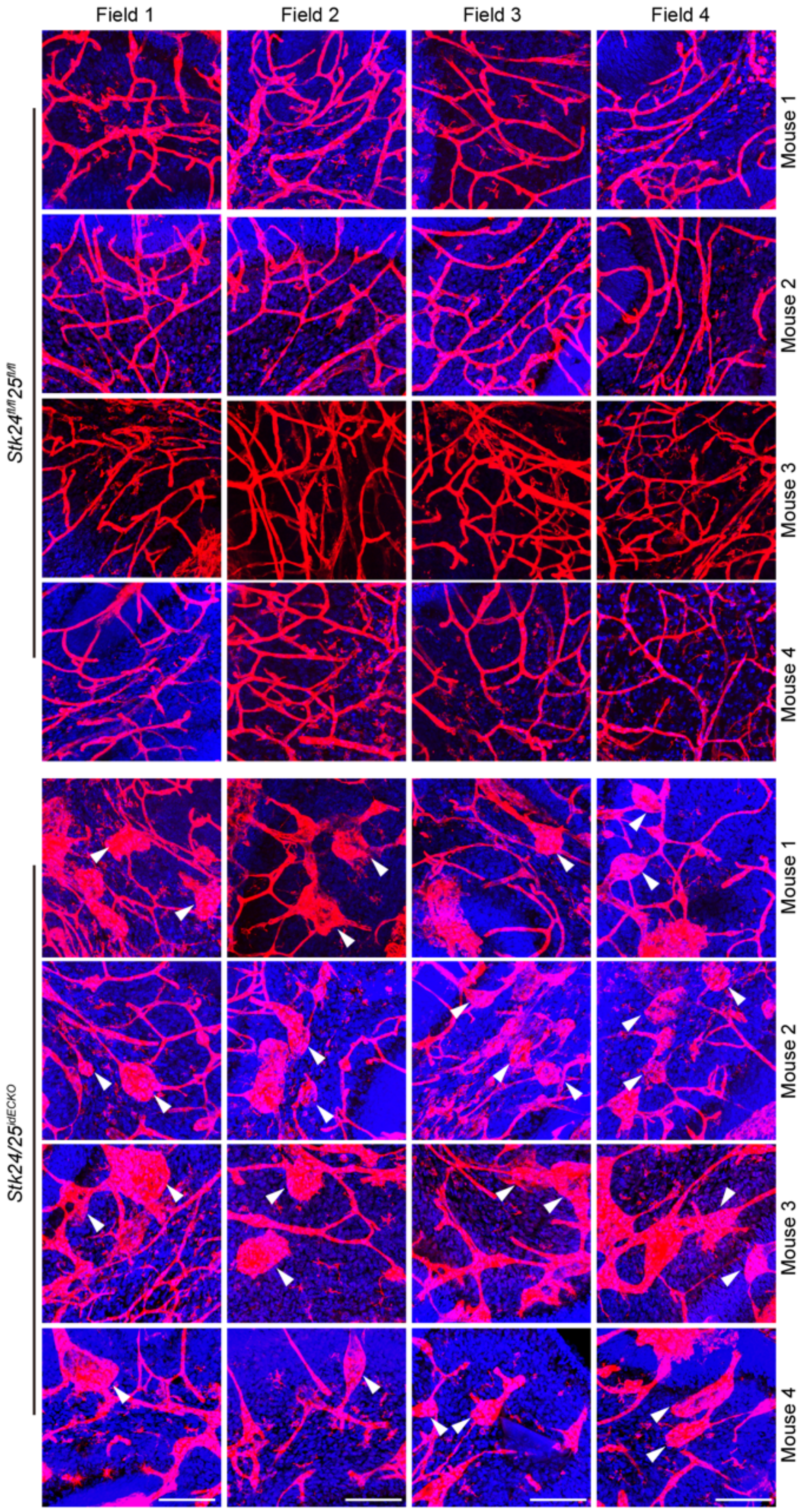

Fig. S6. Deletion of *Stk24* and *Stk25* in endothelium of new born pups resulted in malformed vessels in the cerebellum. Confocal images of IsoB4 staining in the cerebellum vasculature of P8 control and *Stk24/25*^{idECKO} pups. Cavernous vessels in the *Stk24/25*^{idECKO} mice are indicated by the white arrows. Scale bars, 100 µm.

Supplemental Figure 7

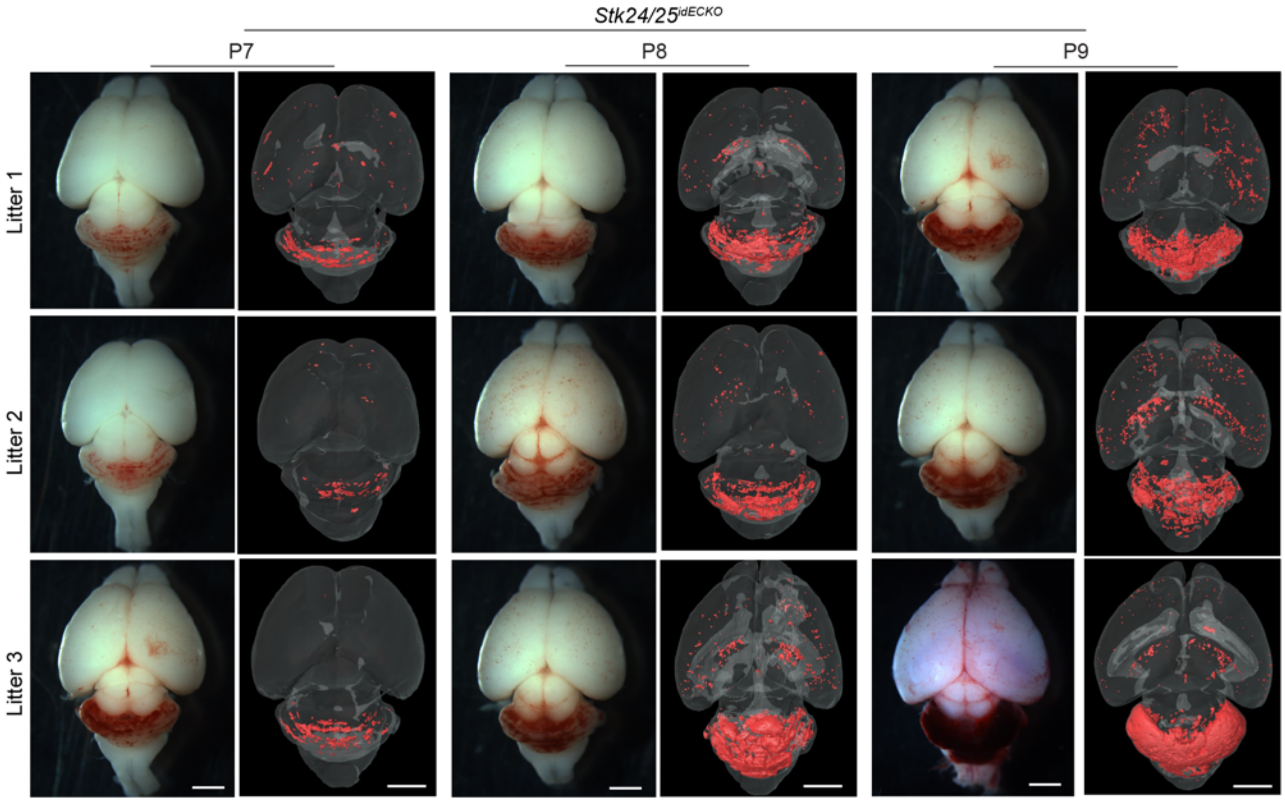

Fig. S7. Progressive lesion development in the *Stk24/25*^{idECKO} mice. Stereomicroscopic images and micro-CT images of CCM lesions in the *Stk24/25*^{idECKO} littermates at P7, P8 and P9 after induction at P2 (n=3 for each time point). Scale bars, 2 mm.

Supplemental Figure 8

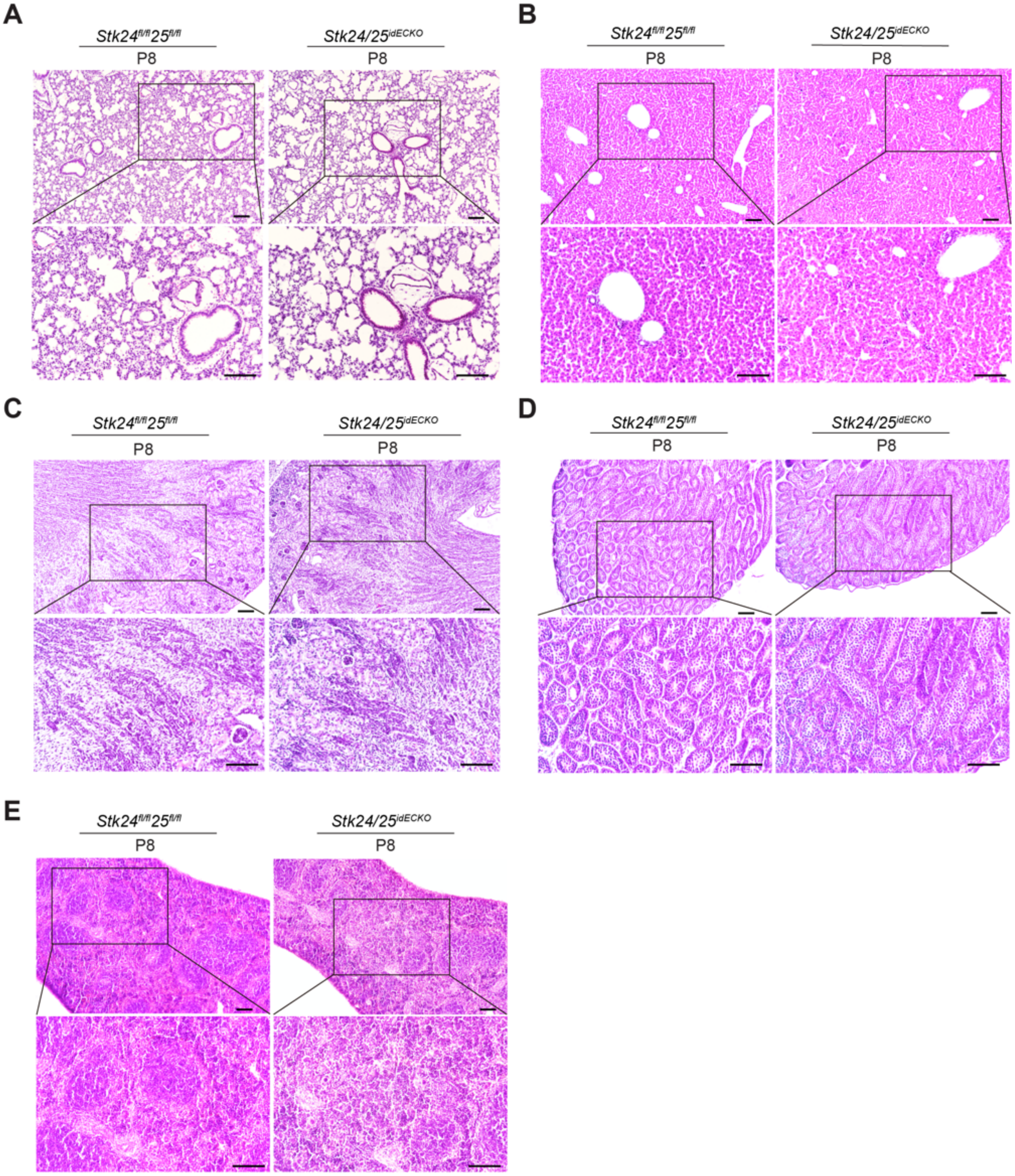

Fig. S8. H&E staining of peripheral organs in the control and *Stk24/25*^{idECKO} mice . H&E staining images of sections of lung (**A**), liver (**B**), kidney (**C**), testis (**D**) and spleen (**E**) show no evidences of hemorrhage or lesions in control and *Stk24/25*^{idECKO} mice. Scale bar, 100 µm.

Supplemental Figure 9

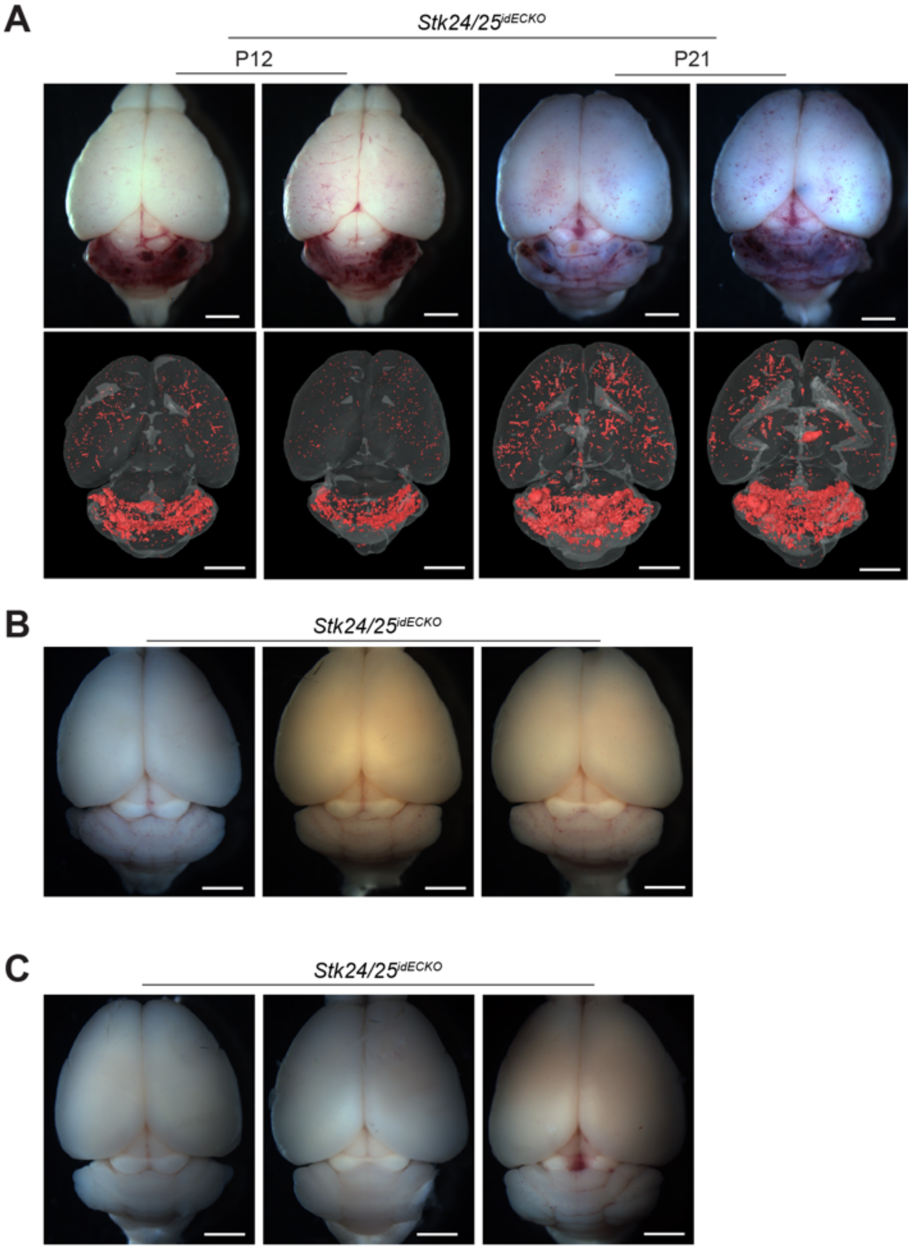

Fig. S9. Limited induction time window of CCM formation in the *Stk24/25*^{idECKO} mice. **A**) Stereomicroscopic images and micro-CT images of CCM lesions in the *Stk24/25*^{idECKO} mice after 4-HT induced deletion at P5. Scale bars, 2 mm. **B**) Stereomicroscopic images and micro-CT images of CCM lesions in *Stk24/25*^{idECKO} mice (P30) after induction at P10, P12 and P14. Scale bars, 2 mm. **C**) Stereomicroscopic images and micro-CT images of CCM lesions in the *Stk24/25*^{idECKO} mice (P30) after induction at P15, P17 and P19. Scale bars, 2 mm.
